# Effects of Anticoagulants and Immune Agents on Pregnancy Outcomes and Offspring Safety in Frozen-Thawed Embryo Transfer Cycles—A Retrospective Cohort Study

**DOI:** 10.3389/fendo.2022.884972

**Published:** 2022-06-21

**Authors:** Yanli Fan, Yizhuo Wang, Zhuoye Luo, Yueming Xu, Jie Zhang, Wei Wang, Na Cui, Guimin Hao

**Affiliations:** Hebei Key Laboratory of Infertility and Genetics, Hebei Clinical Research Center for Birth Defects, Department of Reproductive Medicine, Second Hospital of Hebei Medical University, Shijiazhuang, China

**Keywords:** anticoagulation, immunotherapy, frozen-thawed embryo transfer, congenital anomalies, recurrent miscarriage

## Abstract

The application of anticoagulants and immune agents in assisted reproduction technology has been in a chaotic state, and no clear conclusion has been reached regarding the effectiveness and safety of this treatment. We aimed to explore the potential association between adjuvant medication and pregnancy outcomes and offspring safety in a retrospective cohort study including 8,873 frozen-thawed embryo transfer cycles. The included cycles were divided into three groups according to the drugs used, namely, the routine treatment group (without anticoagulant agents and immune agents), the anticoagulant agent group, and the immunotherapy group. Among normal ovulatory patients, those who used immune agents had a 1.4-fold increased risk of miscarriage (≤13 weeks), but a 0.8-fold decreased chance of birth (**≥**28 weeks) compared with the routine treatment group. Among patients with more than 1 embryo transferred, those who used anticoagulant agents showed a 1.2-fold higher risk of multiple birth than those undergoing routine treatment. Among patients without pregnancy complications, anticoagulant treatment was associated with a 2.1-fold increased risk of congenital anomalies. Among young patients (<26 years) with a singleton pregnancy, the neonatal birth weight of the immunotherapy group and the anticoagulant treatment group was 305.4 g and 175.9 g heavier than the routine treatment group, respectively. In conclusion, adjuvant anticoagulants or immune agent treatment in assisted reproductive technology should be used under strict supervision, and the principle of individualized treatment should be followed.

## 1 Introduction

With the rapid and dramatic development of assisted reproductive technology (ART) (1, 2), the demand of infertile couples for ART has expanded from helping to obtain pregnancy to improving the ongoing pregnancy rate and live birth rate and reducing the miscarriage rate per embryo transfer cycle, reflecting people’s enthusiastic expectation of a high pregnancy rate with a low risk of adverse events. In particular, the etiologies of recurrent miscarriage, repeated implantation failure, and long-term infertility with unknown reasons remain unclear, resulting in the lack of standardized investigation and management. Therefore, numerous adjuvant therapies have been introduced, such as the application of anticoagulants, immunosuppressants, and immunomodulators (3, 4). Due to the lack of strict supervision, the clinical application of such medications lacks standardization, bringing a potential risk of drug abuse. On this issue, some experts suggested that overtreatment should be avoided when prescribing individualized therapy according to couples’ preferences (5). The application of anticoagulants and immune agents in ART has been in a chaotic state (5–7), and no clear conclusion has been reached. Frozen-thawed embryo transfer (FET) cycles are ideal models for investigating the independent effect of adjuvant drugs since the confounding effect of ovarian stimulation is removed. Thus, we aimed to explore the effectiveness and safety of the adjuvant use of anticoagulants and immune agents in this retrospective cohort study on FET cycles.

## 2 Materials and Methods

**Figure 1:**
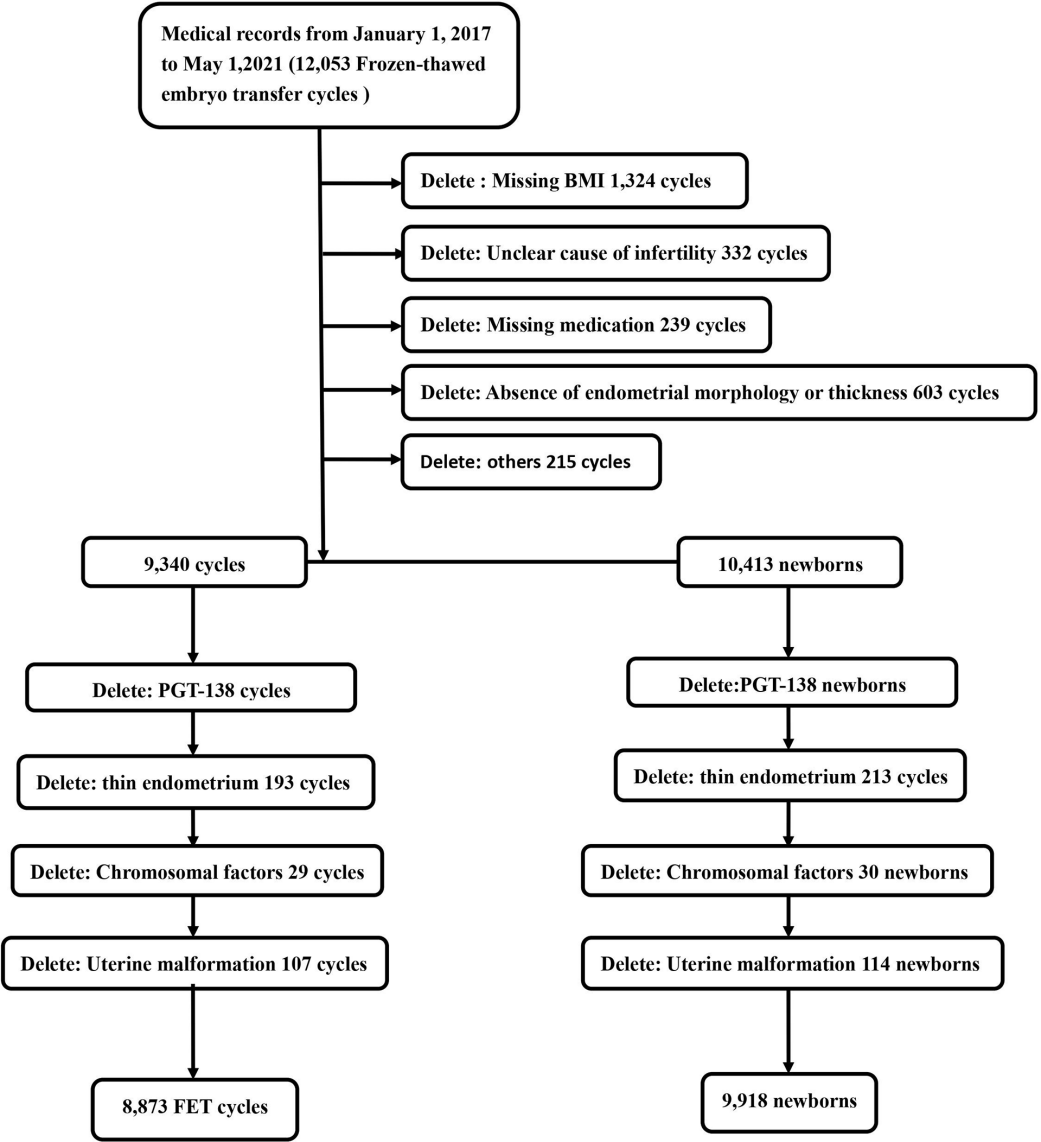
Study flowchart.

This retrospective cohort study was conducted in the Reproductive Medicine Center of the Second Hospital of Hebei Medical University, a tertiary hospital. A total of 12,053 FET cycles from January 1, 2017 to May 1, 2021 were reviewed for eligibility. Women aged 20–49 years who underwent FET were included in this study. Subjects who met any of the following criteria were excluded: (a) thin endometrium (<7 mm, measured at least three times) (8); (b) uterine malformation; (c) preimplantation genetic testing (PGT); (d) missing essential data and information; and (e) chromosome polymorphism. After excluding 3,180 subjects, a total of 8,873 cycles (resulting in 9,918 newborns) with complete data were included in the study (**Figure 1**). The study protocol was approved by the Ethics Committee of the Second Hospital of Hebei Medical University. The Second Hospital of Hebei Medical University provided administrative permission for the research team to access and use the data included in this research.

### 2.1 Cycle Regimens

#### 2.1.1 Hormone Replacement Therapy

The transfer of thawed embryos was carried out when the endometrial thickness reached 8 mm after a step-up regimen for endometrial preparation. Estradiol valerate (Progynova^®^, Bayer) was administered orally at 6–8 mg/day on day 2 of the menstrual cycle, which was followed by vaginal administration of micronized progesterone (Uterogestan, Besins International, France) 400 mg BID or combined administration of oral dydrogesterone (Duphaston^®^; Abbott Biologicals, Netherlands) 10 mg BID and progesterone/oil injection (Progesterone Injection 20 mg/ml, Zhejiang Xianju Pharmaceutical Co., Ltd., China) 40–60 mg QD.

#### 2.1.2 Natural Cycle

A serial ultrasound scan was performed every 2 days from menstrual cycle day 10–12. Once the dominant follicle reached 16–20 mm in diameter, HCG was injected for the trigger of ovulation, and progesterone/oil injection or oral dydrogesterone was prescribed at 40 mg QD or 10 mg BID, respectively, as luteal phase support.

#### 2.1.3 Ovulation Induction Cycle

Letrozole 2.5–5 mg QD was started from menstrual cycle day 2–3, followed by human menopausal gonadotropin (HMG) injections for ovulation induction. The starting dose of HMG (37 or 75 IU) was determined by follicular development. When the dominant follicle reached 18 mm in diameter and the endometrial thickness reached 8 mm, HCG was administered at 10,000 IU for the trigger of ovulation. The transfer of frozen-thawed cleavage-stage embryo was performed 4 days later and luteal phase support was given as described above.

#### 2.1.4 Gonadotropin-Releasing Hormone Agonist Downregulation Combined With Hormone Replacement Therapy

Patients received a single injection of 3.75 mg of long-acting triptorelin acetate on menstrual cycle day 2 after an ultrasound scan confirmed ovarian quiescence and the presence of a thin endometrium (<5 mm). After 28 to 30 days, sequential estrogen and progesterone were prescribed as in the HRT cycles.

### 2.2 Adjuvant Medication

Patients who used aspirin or low-molecular-weight heparin were allocated into the anticoagulant group, while those who used prednisone, hydroxychloroquine, or cyclosporine (whether in combination with anticoagulants or not) were allocated into the immunotherapy group. The remaining patients without anticoagulant and immune agent treatment were allocated into the routine treatment group.

Anticoagulants

Aspirin: Aspirin Enteric-Coated Tablets (Bayer Health Care Manufacturing S.r.l.), 50-75 mg, QD was started from the day of estradiol valerate tablets or progesterone initiation until 10–12 weeks of pregnancy.

Low-molecular-weight heparins: Enoxaparin Sodium Injection (Clexane, Sanofi-Aventis SA, Paris, France) 0.6 ml: 6000AxaIU or Nadroparin Calcium Injection (Fraxiparine, Glaxo Smith Kline, Brentford, UK) 0.4 ml: 4100AxaIU or Low-molecular-weight heparin calcium injection (Aosida, Hebei Changshan, Shijiazhuang, China) 0.4 ml: 4100AxaIU was injected subcutaneously QD from the day of embryo transfer until 6–8 weeks after transfer.

Immune agents

Prednisone (Prednisone Acetate Tables, Xianju, Taizhou, China), 5 mg, QD or Hydroxychloroquine Sulfate tablets (Fenle, SPH zhongxi, Shanghai, China) 0.1 g*14/Hydroxychloroquine Sulfate tablets (Plaquenil, Sanofi-Aventis SA, Paris, France) 0.2 g*10, 0.2 g, BID or Cyclosporine Soft Capsules (New sespin, Zhongmeihuadong Pharmaceutical Co. Hangzhou, China) 50 mg*50, 50 mg, BID were prescribed from the day of embryo transfer until 6–8 weeks after transfer.

### 2.3 Clinical and Birth Outcomes

Two authors independently extracted and reexamined the clinical and birth outcome data from medical records. The diagnosis of clinical outcomes or diseases was made by professional physicians using standardized criteria. Miscarriage was defined as pregnancy loss before 13 weeks of gestation; birth referred to both live birth and stillbirth after 28 weeks of gestation. Preterm delivery was defined as birth between 28 and 37 gestational weeks. Pregnancy complications included hypertensive disorder of pregnancy (HDP) (329 cycles) (9, 10), gestational diabetes mellitus (GDM) (83 cycles) (11), HDP and GDM (13 cycles), premature rupture of membranes (296 cycles) (12), cervical insufficiency (20 cycles) (13), oligohydramnios (28 cycles) (14), polyhydramnios (6 cycles) (15), placenta previa (36 cycles) (16), abruptio placentae (7 cycles) (17), postpartum hemorrhage (6 cycles) (18), disseminated hematogenous tuberculosis (1 cycle) (19), intrahepatic cholestasis of pregnancy (2 cycles) (20), postpartum thrombotic disease (3 cycles) (21), chronic nephritis (1 cycle), and unclear diagnosis (45 cycles). The categorization of pregnancy complications is shown in **Table S2** as **Supplemental Material**. The diagnosis criteria for the above-mentioned pregnancy complications were reported previously.

There were 85 cases of congenital anomalies involving six major systems; 7 cases were found to have chromosomal abnormalities or abnormal nuchal translucency and 4 cases were without clear description. Detailed information is shown in **Table S1** as **Supplemental Material**.

### 2.4 Statistical Analysis

Continuous variables were presented as median (interquartile range) or mean ± standard deviation (SD) according to the normality of distribution. Kruskal–Wallis test was used to compare the continuous variables among the three groups. Categorical variables were presented as count (percentage) and compared using the chi-square test. Logistic regression analysis and stratified analysis were used to explore the associations between adjuvant medication and miscarriage, birth, multiple birth, congenital anomaly, and birth weight with the generalized estimation equation (GEE) model to deal with the repeat cycles and data of twins. Two regression models were applied: baseline characteristics of study subjects in model 1, while other confounding factors were adjusted in model 2. Confounders on the basis of their associations with the outcomes of interest or a change in effect estimate of more than 10% were selected. All the analyses were performed using R 3.6.3 (http://www.r-project.org) and EmpowerStats (www.empowerstats.net, X&Y Solutions Inc., Boston, MA, USA), and a two-sided *p*-value of <0.05 was considered to indicate statistical significance.

## 3 Results

### 3.1 Baseline Characteristics, Laboratory Data, Pregnancy, and Neonatal Outcomes of Study Subjects

As shown in **Table 1**, there were 8,873 FET cycles included in this retrospective study, among which 4,253 cycles were allocated into the routine treatment group, 3,698 cycles were allocated into the anticoagulant group, and 922 were allocated into the immunotherapy group.

**Table 1:** Patient characteristics (8,873 FET cycles).

	Routine treatment	Anticoagulant treatment		Immunotherapy	
*N* (cycles)	4,253	3,698		922	
	Mean ± SD/*N* (%)	Mean ± SD/*N* (%)	*p*	Mean ± SD/*N* (%)	*p*
**Age—Female (years)**	30.6 ± 4.3	30.7 ± 4.3	0.248	31.4 ± 4.3	<0.001
**Age—Male (years)**	31.4 ± 4.8	31.5 ± 4.7	0.407	32.2 ± 5.0	<0.001
**Infertility Duration (years)**	4.4 ± 3.0	4.5 ± 3.0	0.192	4.6 ± 2.8	0.055
**BMI**	23.4 ± 3.6	23.5 ± 3.6	0.301	23.3 ± 3.4	0.519
**Cycle Number**	1.5 ± 0.8	1.4 ± 0.8	0.014	2.0 ± 1.1	<0.001
**Endometrial Thickness (mm)**	9.7 ± 1.5	9.6 ± 1.5	0.008	9.6 ± 1.5	0.033
**No. of Transferred Embryos**	1.8 ± 0.4	1.8 ± 0.4	/	1.6 ± 0.5	<0.001
**Infertility (type)**			0.549		0.049
Primary infertility	2,200 (51.7%)	1,888 (51.1%)		444 (48.2%)	
Secondary infertility	2,053 (48.3%)	1,810 (48.9%)		478 (51.8%)	
**Etiology of Infertility**			0.026		0.125
Ovulation disorders★	396 (9.3%)	400 (10.8%)		101 (11.0%)	
Other	3,857 (90.7%)	3,298 (89.2%)		821 (89.0%)	
**Previous Miscarriage**			0.441		<0.001
0	3,002 (70.6%)	2,563 (69.3%)		582 (63.1%)	
1–2	1,126 (26.5%)	1,026 (27.7%)		313 (33.9%)	
≥3	125 (2.9%)	109 (2.9%)		27 (2.9%)	
**FET Protocol**			<0.001		<0.001
NC	643 (15.1%)	74 (2.0%)		43 (4.7%)	
OI	180 (4.2%)	42 (1.1%)		45 (4.9%)	
HRT	2,980 (70.1%)	3,039 (82.2%)		674 (73.1%)	
GnRHa-HRT	450 (10.6%)	543 (14.7%)		160 (17.4%)	
**Endometrial Echogenicity**			0.812		<0.001
A	2,791 (65.6%)	2,433 (65.8%)		453 (49.1%)	
B	1,442 (33.9%)	1,244 (33.6%)		462 (50.1%)	
C	20 (0.5%)	21 (0.6%)		7 (0.8%)	
**Developmental Stage of the Transferred Embryo**			0.025		<0.001
Cleavage-stage embryo	3,148 (74.0%)	2,653 (71.7%)		448 (48.6%)	
Blastocyst	1,085 (25.5%)	1,034 (28.0%)		473 (51.3%)	
Sequential transfer of cleavage stage Embryo and blastocyst	20 (0.5%)	11 (0.3%)		1 (0.1%)	
**Pregnancy Location**			0.341		0.837
No	1,980 (46.6%)	1,652 (44.7%)		436 (47.3%)	
Intrauterine pregnancy	2,213 (52.0%)	1,999 (54.1%)		476 (51.6%)	
Ectopic pregnancy	51 (1.2%)	40 (1.1%)		9 (1.0%)	
Heterotopic pregnancy	9 (0.2%)	7 (0.2%)		1 (0.1%)	
**Early Miscarriage (≤13 weeks)**			0.285		0.007
No	1,903 (83.7%)	1,688 (82.5%)		382 (78.6%)	
Yes	370 (16.3%)	358 (17.5%)		104 (21.4%)	
**Birth (**≥**28 weeks)**			0.504		0.020
No	424 (18.7%)	398 (19.5%)		113 (23.3%)	
Yes	1,849 (81.3%)	1,648 (80.5%)		373 (76.7%)	
**Multiple Birth**			0.418		0.008
No	1,361 (73.6%)	1,193 (72.4%)		299 (80.2%)	
Yes	488 (26.4%)	455 (27.6%)		74 (19.8%)	
**Gestational Weeks at Birth**			0.891		0.708
Preterm	406 (22.0%)	363 (22.0%)		79 (21.2%)	
Term	1,441 (77.9%)	1,284 (77.9%)		293 (78.6%)	
Postterm	2 (0.1%)	1 (0.1%)		1 (0.3%)	
**Pregnancy Complications**			0.662		0.934
No	3,817 (90.2%)	3,294 (89.9%)		825 (90.3%)	
Yes	416 (9.8%)	371 (10.1%)		89 (9.7%)	
**Cycles (categorized)**			0.027		<0.001
1	2,838 (66.7%)	2,570 (69.5%)		359 (38.9%)	
2	998 (23.5%)	785 (21.2%)		330 (35.8%)	
≥3	417 (9.8%)	343 (9.3%)		233 (25.3%)	

★Ovulation disorders refers to the patients with ovulation disorder of group II and III as defined by WHO (22).

FET, frozen-thawed embryo transfer; BMI, body mass index; NC, natural cycle; OI, ovulation induction; HRT, hormone replacement therapy; GnRHa-HRT, gonadotropin-releasing hormone (GnRH) agonist downregulation combined with hormone replacement therapy.

As for the baseline characteristics of the study population, the age of couples in the immunotherapy group was greater than those in the routine treatment group (female: 31.4 ± 4.3 vs. 30.6 ± 4.3, *p* < 0.001; male: 32.2 ± 5.0 vs. 31.4 ± 4.8, *p* < 0.001). Patients in the immunotherapy group had longer infertility duration (4.6 ± 2.8 vs. 4.4 ± 3.0, *p* = 0.055), higher proportion of patients with 1–2 previous miscarriages [313 (33.9%) vs. 1,126 (26.5%), *p* < 0.001], and more repetition cycles (≥3) [233 (25.3%) vs. 417 (9.8%), *p* < 0.001], but the proportion of infertility type, etiology of infertility, and body mass index (BMI) was comparable between the two groups. Compared with the routine treatment group, the anticoagulant treatment group had a higher proportion of patients with 1 cycle [2,570 (69.5%) vs. 2,838 (66.7%), *p* = 0.027] and more patients with ovulation disorders [400 (10.8%) vs. 396 (9.3%), *p* = 0.026], while the age of couples, infertility duration, BMI, infertility type, and previous miscarriages were comparable between the two groups.

In terms of laboratory variables, the routine treatment group had a higher proportion of cleavage-stage embryo transfer [3,148 (74.0%) vs. 2,653 (71.7%) vs. 448 (48.6%)], while the immunotherapy group had a higher proportion of blastocyst transfer [473 (51.3%) vs. 1,085 (25.5%) vs. 1,034 (28.0%)]. As for pregnancy outcomes, the immunotherapy group demonstrated a higher miscarriage rate [104 (21.4%) vs. 370 (16.3%) vs. 358 (17.5%)] but a lower multiple birth rate [74 (19.8%) vs. 488 (26.4%) vs. 455 (27.6%)] and birth rate [373(76.7%) vs.1,849 (81.3%)vs.1,648 (80.5%)). However, there was no significant difference in the gestational weeks at birth, pregnancy location, and pregnancy complications among the three groups.

Neonatal outcomes are shown in **Table 2**, including 9,918 newborns. There were 4,758 newborns in the routine medication group, 4,164 newborns in the anticoagulant treatment group, and 996 newborns in the immunotherapy group. No significant differences were found in congenital anomaly and gender, while the immunotherapy group had greater neonatal weight (3,031.3 ± 684.2 vs. 2,960.9 ± 692.7 vs. 2,965.5 ± 678.4, *p* = 0.048).

**Table 2:** Neonatal characteristics (9,918 neonates).

	Routine treatment	Anticoagulant treatment		Immunotherapy	
N	4,758	4,164		996	
	Mean ± SD/*N* (%)	Mean ± SD/*N* (%)	*p*	Mean ± SD/*N* (%)	*p*
**Neonatal weight (g)**	2,960.9 ± 692.7	2,965.5 ± 678.4	0.824	3,031.3 ± 684.2	0.048
**Gender**			0.355		0.302
Female	1,221 (51.6%)	1,125 (53.0%)		243 (54.2%)	
Male	1,146 (48.4%)	999 (47.0%)		205 (45.8%)	
**Congenital anomaly**			0.396		0.319
No	4,714 (99.1%)	4,118 (98.9%)		990 (99.4%)	
Yes	44 (0.9%)	46 (1.1%)		6 (0.6%)	

### 3.2 Adjuvant Medications Were Associated With Inferior Pregnancy Outcomes by Multivariate Regression Analysis With Stratification

Multivariate regression analysis with stratification was used to investigate the effectiveness of adjuvant medication on improving pregnancy outcomes. After adjusting for age of the couples, BMI, infertility duration, the number of transferred embryos, endometrial echogenicity, FET protocol, number of previous miscarriage, and cycle number, normal ovulatory patients undergoing immunotherapy demonstrated a 40% (OR = 1.4, 95% CI: 1.0, 1.8) higher risk of miscarriage (**Table 3**) and a 20% (OR = 0.8, 95% CI: 0.6, 1.0) lower probability of birth (**Table 3**) compared with those without adjuvant medication. Moreover, patients with more than 1 embryo transferred and anticoagulant treatment showed an increased risk of multiple birth (OR = 1.2, 95% CI: 1.0, 1.4) after controlling for confounding factors including age of the couples, BMI, infertility duration, endometrial thickness, cycle number, and the number of previous miscarriage (**Table 4**).

**Table 3:** Multivariate logistic regression of miscarriage and birth (≥28 weeks) stratified by the etiology of infertility among patients undergoing routine treatment, anticoagulant treatment, and immunotherapy.

	N	Routine treatment	Anticoagulant treatment		Immunotherapy	
		Reference	OR (95% CI)	P	OR (95%CI)	p
**Y=miscarriage**						
Etiology of Infertility						
OD	529	1.0	0.8 (0.5, 1.4)	0.414	0.6 (0.3, 1.5)	0.302
Other	4,276	1.0	1.1 (0.9, 1.3)	0.441	1.4 (1.0, 1.8)	0.021
**Y = birth (≥28 weeks)**						
Etiology of Infertility						
OD	529	1.0	1.3 (0.8, 2.1)	0.385	1.7 (0.7, 3.9)	0.230
Other	4,276	1.0	1.0 (0.8, 1.1)	0.710	0.8 (0.6, 1.0)	0.032

OD, ovulation disorder.

Adjusted for Age—female, Age—male, BMI, Infertility duration, No. of transferred embryos, Endometrial echogenicity, FET protocol, Previous miscarriage, and Cycle number.

**Table 4:** Multivariate logistic regression of multiple birth stratified by the number of transferred embryos among patients undergoing routine treatment, anticoagulant treatment, and immunotherapy.

Y = Multiple birth		Routine treatment	Anticoagulant treatment		Immunotherapy	
No. of transferred embryos	*n*	Reference	OR (95% CI)	*P*	OR (95% CI)	*p*
1	785	1.0	0.6 (0.2, 1.8)	0.286	1.0 (0.3, 3.5)	0.971
>1	3,085	1.0	1.2 (1.0, 1.4)	0.021	1.0 (0.7, 1.3)	0.880

Adjusted for Age—female, Age—male, BMI, Infertility duration, Endometrial thickness, Previous miscarriage, and Cycle number.

### 3.3 Adjuvant Medications Significantly Impact Offspring Safety by Multivariate Regression Analysis With Stratification

After controlling for gestational weeks at birth, multiple birth, age of the couples, BMI, developmental stage of transferred embryos, infertility type, the number of embryos transferred, and cycle number, neonates of patients without pregnancy complications but undergoing anticoagulant therapy showed an increased risk of congenital anomalies (adjusted OR = 2.1, 95% CI: 1.0, 4.5) (**Table 5**). However, in patients with pregnancy complications, the risk of congenital anomaly was comparable among the three groups.

**Table 5:** Univariate and multivariate logistic regression of congenital anomaly stratified by pregnancy complications among patients undergoing routine treatment, anticoagulant treatment, and immunotherapy.

Y = Congenital Anomalies	Without pregnancy complications		With pregnancy complications		Total	
	OR (95% CI)	*p*	OR (95% CI)	*p*	OR (95% CI)	*p*
**Non-adjusted**						
Routine treatment^a^	1.0		1.0		1.0	
Anticoagulant treatment	1.9 (1.1, 3.4)	0.022	0.6 (0.3, 1.2)	0.121	1.2 (0.8, 1.8)	0.470
Immunotherapy	1.0 (0.3, 3.0)	0.992	0.4 (0.1, 1.8)	0.228	0.7 (0.3, 1.6)	0.372
**Model I**						
Routine treatment	1.0		1.0		1.0	
Anticoagulant treatment	1.9 (1.1, 3.4)	0.021	0.6 (0.3, 1.2)	0.132	1.2 (0.8, 1.8)	0.493
Immunotherapy	0.9 (0.3, 2.6)	0.881	0.5 (0.1,2.0)	0.320	0.7 (0.3, 1.6)	0.358
**Model II**						
Routine treatment	1.0		1.0		1.0	
Anticoagulant treatment	2.1 (1.0, 4.5)	0.046	0.6 (0.3, 1.4)	0.234	1.1 (0.7, 1.9)	0.629
Immunotherapy	0.9 (0.2, 4.8)	0.946	0.6 (0.1, 2.5)	0.498	0.7(0.2, 2.1)	0.528

Non-adjusted model adjust for: None.

Model I adjusted for Age—female, Age—male, BMI, and Cycle Number.

Model II adjusted for Age—female, Age—male, BMI, Gestational weeks at birth, No. of transferred embryos, Developmental stage of transferred embryos, Multiple birth, and Cycle Number.

^a^Routine treatment group served as the reference.

Given the significant influence of maternal age on neonatal birth weight, stratification by female age was performed when investigating the association between adjuvant medication use and neonatal birth weight. The results showed that, among young patients (<26 years) (23) with a singleton pregnancy, the neonatal birth weight of the immunotherapy group was 305.4 g heavier than the routine treatment group (adjusted *β* = 305.4; 95% CI: 55.2, 555.5), while that of the anticoagulant treatment group was 175.9 g heavier than the routine treatment group (adjusted *β* = 175.9, 95% CI, 68.1, 283.7) after adjusting for gestational weeks at birth, male age, BMI, developmental stage of transferred embryos, cycle number, infertility type, and the number of embryos transferred (**Table 6**). In other age strata and among patients with multiple pregnancy, the neonatal birth weight was comparable among the three groups.

**Table 6:** Univariate and multivariate linear regression of birth weight stratified by maternal age among singletons and non-singletons.

Y = Birth Weight (g)	Singleton							
	<26 years		≥26, <38 years		≥38 years		Total	
	β (95% CI)	*p*	β (95% CI)	*p*	β (95% CI)	*p*	β (95% CI)	*p*
**Non-adjusted**								
Routine treatment^a^	0		0		0		0	
Anticoagulant treatment	200.3 (50.2, 350.5)	0.009	−11.6 (−69.8, 46.6)	0.696	−172.8 (−435.6, 89.9)	0.197	2.2 (−51.2, 55.6)	0.936
Immunotherapy	382.9 (119.3, 646.4)	0.004	−26.7 (−106.3, 52.9)	0.511	−37.4 (−293.2, 218.4)	0.775	9.2 (−65.2, 83.7)	0.808
**Model I**								
Routine treatment	0		0		0		0	
Anticoagulant treatment	210.1 (55.3, 365.0)	0.008	−10.7 (−68.9, 47.5)	0.718	−235.9 (−491.8, 20.0)	0.071	3.0 (−50.4, 56.5)	0.911
Immunotherapy	351.1(77.1, 625.0)	0.012	−29.6 (−110.6, 51.5)	0.475	−90.8 (−339.1, 157.4)	0.473	4.0 (−72.0, 80.1)	0.917
**Model II**								
Routine treatment	0		0		0		0	
Anticoagulant treatment	175.9 (68.1, 283.7)	0.001	−14.0 (−53.2, 25.2)	0.484	−175.4 (−354.5, 3.8)	0.055	−1.1 (−37.2, 35.0)	0.951
Immunotherapy	305.4 (55.2, 555.5)	0.017	−48.6 (−110.2, 13.1)	0.123	−159.9 (−406.6, 86.8)	0.204	−21.6 (−80.8, 37.5)	0.473
	**Non-singleton**							
**Non-adjusted**								
Routine treatment^a^	0		0		0		0	
Anticoagulant treatment	−8.8 (−174.0, 156.3)	0.916	39.7 (−22.5, 101.8)	0.211	40.9 (−384.1, 465.9)	0.851	33.6 (−24.0, 91.2)	0.253
Immunotherapy	30.5 (−151.7, 212.8)	0.743	−8.7 (−152.8, 135.4)	0.906	110.5 (−259.6, 480.6)	0.558	−6.8 (−143.1, 129.5)	0.922
**Model I**								
Routine treatment	0		0		0		0	
Anticoagulant treatment	−13.0 (−178.4, 152.4)	0.878	43.6 (−19.6, 106.7)	0.177	−36.8 (−461.8, 388.2)	0.865	36.8 (−21.5, 95.1)	0.216
Immunotherapy	63.8 (−174.3, 301.8)	0.600	−6.3 (−145.3, 132.7)	0.929	169.6 (−171.2, 510.5)	0.329	−9.1 (−140.3, 122.2)	0.892
**Model II**								
Routine treatment	0		0				0	
Anticoagulant treatment	−40.5 (−182.8, 101.7)	0.576	33.4 (−20.3, 87.2)	0.223			23.7 (−25.3, 72.8)	0.344
Immunotherapy	17.2 (−189.9, 224.3)	0.870	20.0 (−104.8, 144.8)	0.753			2.3 (−115.5, 120.1)	0.970

Non-adjusted model adjusted for: None

Model I adjusted for Age—male, BMI, and Cycle Number.

Model II adjusted for Age—male, BMI, Gestational weeks at birth, No. of transferred embryos, Developmental stage of transferred embryos, and Cycle Number.

^a^Routine treatment group served as the reference.

## Discussion

In this retrospective cohort study of 8,873 FET cycles (9,918 newborns), we observed that anticoagulation and immunotherapy had a significant influence on pregnancy outcomes and offspring safety.

Compared with the routine treatment group, using immune agents was associated with an increased risk of miscarriage and a decreased rate of birth in normal ovulatory patients. A fetus has antigens of maternal and paternal origins (5). The physiological mechanisms of the immunotolerance of paternal antigens during pregnancy are poorly understood. However, a dysfunction in immune modulation has been hypothesized to be one of the causes of infertility or miscarriage. Several systematic reviews (24–26) have evaluated the effectiveness and safety of immunological interventions for recurrent miscarriage, and none of such interventions were associated with a reduction in miscarriages or an increase in live births. Thus, there was insufficient evidence to recommend immunotherapy in the management of recurrent miscarriage. In this study, we found that the adjuvant immunotherapy during FET cycles significantly increased the risk of miscarriage, but markedly decreased the probability of birth among normal ovulatory patients only. In contrast, an increase in birth and a decrease in miscarriage were witnessed among non-ovulatory patients undergoing immunotherapy, although both were without statistical significance. This suggested that patients with ovulation disorder may have underlying defects in immunomodulation during embryo implantation, so that they can benefit from immunotherapy. However, among patients with normal ovulation, the administration of exogenous immune agents may in turn disturb their immunotolerance to fetal antigen, resulting in an increased miscarriage rate and a decreased birth rate.

Using anticoagulant agents was associated with a higher risk of multiple deliveries and an increased risk of congenital anomalies. In terms of anticoagulant therapy, several systematic reviews and meta-analyses (4, 27–29) have shown that low-dose aspirin and low-molecular-weight heparin could effectively reduce the miscarriage rate and increase the live birth rate in women with antiphospholipid syndrome or a history of recurrent miscarriage. The combination of low-molecular-weight heparin and aspirin during pregnancy may increase the live birth rate in women with persistent anti-phospholipid (aPL) when compared with aspirin treatment alone. In this study, anticoagulant therapy significantly increased twin birth rate in patients with more than 1 embryo transferred. Our finding is consistent with the published studies suggesting the improvement in live birth rate by using anticoagulation therapy. However, there were few articles focusing on the relationship between anticoagulation therapy and congenital malformations. A randomized controlled trial reported few cases of congenital anomalies, but this may be underestimated given the small sample size (30). Our study collected the clinical data of 9,918 neonates, among which 96 cases of congenital anomalies were observed. We classified fetal congenital anomalies according to the human body system (**Table S1**). There were 44 cases in the routine treatment group, 46 cases in the anticoagulant group, and 6 cases in the immunotherapy group. In the anticoagulant group, 44 cases were exposed to aspirin and 2 cases were exposed to both aspirin and low-molecular-weight heparin, suggesting that aspirin was associated with congenital anomalies. Aspirin can inhibit prostaglandin synthesis and subsequent reduction of platelet aggregation by inactivating cyclooxygenase (31). According to the latest guideline on low-dose aspirin use during pregnancy by the American College of Obstetricians and Gynecologists (ACOG), low-dose aspirin use during the first and the second trimester was considered to be effective and safe (32). In this study, the incidence of congenital anomalies was comparable among the three groups in patients with pregnancy complications, which was consistent with the ACOG guideline. However, in patients without comorbidities during pregnancy, the risk of fetal malformation increased when adjuvant anticoagulants were prescribed. The relationship between aspirin and genitourinary abnormalities and gastroschisis has been reported (33–36). In our study, there were four cases of genitourinary abnormalities: two cases of cryptorchidism (one exposed to aspirin, while the other was from the routine treatment group), one case of hypospadias (from the routine treatment group), and one case of gastroschisis (from the routine treatment group). In addition, after excluding cases with parental chromosomal abnormalities, malformations of systems other than the genitourinary system and gastrointestinal systems were also reported, which is interesting and unexpected. Furthermore, high-quality prospective studies and comprehensive neonatal physical examination are warranted to evaluate the safety of aspirin in the field of reproduction.

In terms of the effect of adjuvant medication on neonatal birth weight, previous systematic reviews only focused on fetal growth restriction and no firm conclusions were drawn (4, 7). In this study, the neonatal birth weight of each group was quantitatively analyzed, and multivariate linear regression was performed to adjust for confounding factors. The results showed that there was a statistically significant increase in neonatal birth weight after the adjuvant use of either anticoagulants or immune agents among patients under the age of 26. This is different from previous reports that fetal weight increases with maternal age (37, 38), suggesting that anticoagulation combined with or without immunotherapy has a positive impact on birth weight.

To summarize, using immune agents was associated with an increased risk of miscarriage and a decrease in birth among normal ovulatory patients. Using anticoagulant agents was associated with a higher risk of multiple birth and an increased risk of congenital anomalies. Young mothers had heavier newborns after either anticoagulant agent or immune agent treatment during FET cycles. Therefore, adjuvant anticoagulant or immune agent treatment in ART should be used under strict supervision, and the principle of individualized treatment should be followed.

## Data Availability Statement

The raw data supporting the conclusions of this article will be made available by the authors, without undue reservation.

## Ethics Statement

The studies involving human participants were reviewed and approved by the Ethics Committee of Second Hospital of Hebei Medical University. Written informed consent for participation was not required for this study in accordance with the national legislation and the institutional requirements.

## Author Contributions

YF, YW, and GH devised the idea and designed the study. ZL and JZ contributed to the primary data collection. YX and JZ reexamined the data and analyzed the data. YF wrote the original draft, which was revised by GH. WW and NC supervised the study and administered the project. All authors contributed to the article and approved the submitted version.

## Funding

This study was supported by the Natural Science Foundation of Hebei Province (H2021206377), S&T Program of Hebei (21377760D), Clinical Medicine Outstanding Talents Program of Government Funds, and Tracking Project of Hebei Province Medical Applicable Technology (GZ2022019). The funding sponsors had no role in the study design; in the collection, analyses, and interpretation of data; in the writing of the manuscript; or in the decision to publish the results.

## Conflict of Interest

The authors declare that the research was conducted in the absence of any commercial or financial relationships that could be construed as a potential conflict of interest.

## Publisher’s Note

All claims expressed in this article are solely those of the authors and do not necessarily represent those of their affiliated organizations, or those of the publisher, the editors and the reviewers. Any product that may be evaluated in this article, or claim that may be made by its manufacturer, is not guaranteed or endorsed by the publisher.

## Abbreviations

FET, frozen-thawed embryo transfer; PGT, preimplantation genetic testing; HRT, hormone replacement therapy; PIO, progesterone in oil injection; NC, natural cycle; OI, Ovulation Induction; GnRHa-HRT, gonadotropin-releasing hormone agonist downregulation combined with hormone replacement therapy; aPL, anti-phospholipid; ART, assisted reproduction technology; HDP, hypertensive disorder of pregnancy; GDM, gestational diabetes mellitus.
