# Supplementary material for: Effects of Anticoagulants and Immune Agents on Pregnancy Outcomes and Offspring Safety in Frozen-Thawed Embryo Transfer Cycles—A Retrospective Cohort Study
Source: Front Endocrinol (Lausanne). 2022 Jun 21;13:884972. doi: 10.3389/fendo.2022.884972 (PMC9255591; doi:10.3389/fendo.2022.884972)
Supplement: Supplementary file 1 [file Table_1.docx]

**Supplemental Materials**

**Table S1:** Congenital Anomalies

| Circulatory System | N=27 |  |
| --- | --- | --- |
| Mitral insufficiency | 1 |  |
| Congenital heart disease(Uncategorized) | 11 | **** |
| Ventricular septal defect | 2 | * |
| Transposition of pulmonary artery | 1 | * |
| Patent foramen ovale | 3 | * |
| Myocardiopathy | 1 |  |
| Chylothorax | 1 | * |
| Cystic hygroma | 1 | * |
| Hemangioma(skin) | 6 | *** |
| **Respiratory System** | **N=3** |  |
| Pulmonary dysplasia | 3 | ★ |
| **Nervous System** | **N=23** |  |
| Retinal dysplasia | 5 | * |
| Hearing impairment | 4 | * |
| Ocular dysplasia | 2 | * |
| Glaucoma | 1 | * |
| Neural tube defects | 1 | * |
| Holoprosencephaly | 1 | ★ |
| Spina bifida | 2 | * |
| Cerebellar vermis dysplasia | 1 |  |
| Hydrocephalus | 6 | **** |
| **Genitourinary system** | **N=3** |  |
| Hypospadias | 1 |  |
| Cryptorchidism | 2 | * |
| **Musculoskeletal System** | **N=12** |  |
| Polydactyly | 2 | * |
| Myasthenia | 2 | ** |
| Limb deformity | 3 | * |
| Congential muscular torticollis | 3 | ** |
| Laryngeal cartilage dysplasia | 1 | * |
| Ear dysplasia | 1 | * |
| **Digestive System** | **N=17** |  |
| Cheilopalatognathus | 4 | *** |
| Cleft palate | 1 | * |
| Congenital intestinal obstruction | 2 | ** |
| Upper gastrointestinal obstruction | 1 |  |
| Mesenteric cyst | 1 |  |
| Imperforate anus | 1 | * |
| Duodenal stenosis | 1 | * |
| Gastroschisis | 1 |  |
| Pyloric obstruction | 1 | * |
| Diaphragmatic hernia | 2 |  |
| Omphalocele | 2 | * |
| **Chromosomal Abnormalities / Abnormal Nuchal Translucency (NT)** | **N=7** |  |
| Chromosome abnormality | 4 |  |
| Abnormal nuchal translucency | 2 | * |
| Hereditary metabolic diseases | 1 |  |
| **Uncategorized** | **N=4** | ****** |

* Exposure to aspirin

★Exposure to aspirin and low molecular weight heparin

**Table S2:** The details of pregnancy complications (except for Chronic Nephritis because of its unclear pathological classification )

| **Pregnancy Complications** | **Cycles** | **Fetus** |  |
| --- | --- | --- | --- |
| Hypertensive Disorder of Pregnancy (18,19) | 329 | 447 |  |
| Gestational Diabetes Mellitus(8) | 83 | 94 |  |
| Hypertensive Disorder of Pregnancy and Diabetes Mellitus | 13 | 17 |  |
| Premature Rupture of Membranes(20) | 296 | 431 |  |
| Cervical Insufficiency(21) | 20 | 29 |  |
| Oligohydramnios(22) | 28 | 30 |  |
| Placenta Previa(24) | 36 | 44 |  |
| Abruptio Placentae(25) | 7 | 8 |  |
| Polyhydramnios(23) | 6 | 9 |  |
| Postpartum Hemorrhage(26) | 6 | 9 |  |
| Disseminated Hematogenous Tuberculosis (27) | 1 | 1 |  |
| Intrahepatic Cholestasis Of Pregnancy(28) | 2 | 4 |  |
| Postpartum Thrombotic Disease(29) | 3 | 4 |  |
| Chronic Nephritis | 1 | 1 |  |
| Unclear Diagnosis | 45 | 57 |  |
| Total | 876 | 1185 |  |
